# Commercial Determinants of Latinx Health: A Scoping Review of Sugar-Sweetened Beverages in the USA

**DOI:** 10.3390/ijerph23060766

**Published:** 2026-06-06

**Authors:** Megan M. Patton-Lopez, Mariana Pinto-Alvarez, Elisa Rivero, Julia Ma, Ileana Carrión, Eric Toole, Daniel F. López-Cevallos

**Affiliations:** 1 Department of Nutrition, School of Public Health and Health Sciences, University of Massachusetts Amherst, Amherst, MA 01003, USA; 2 Department of Health Promotion and Policy, School of Public Health and Health Sciences, University of Massachusetts Amherst, Amherst, MA 01003, USA; mpintoalvare@umass.edu (M.P.-A.); erivero@umass.edu (E.R.); icarrion@umass.edu (I.C.); dlopezcevall@umass.edu (D.F.L.-C.); 3 Department of Psychological and Brain Sciences, School of Natural Sciences, University of Massachusetts Amherst, Amherst, MA 01003, USA; juliama@umass.edu; 4 University Libraries, University of Massachusetts Amherst, Amherst, MA 01003, USA; etoole@umass.edu

**Keywords:** commercial determinants of health, sugar-sweetened beverages, Latinx populations, USA

## Abstract

**Highlights:**

**Public health relevance—How does this work relate to a public health issue?**

- A commercial determinant of health (CDoH) framework is relevant to understand how corporate practices influence Latinx health;
- Sugar-sweetened beverage (SSB) consumption is linked to a range of negative health outcomes among Latinos, including type 2 diabetes and cardiovascular disease.

**Public health significance—Why is this work of significance to public health?**

- Understanding how commercial determinants are impacting Latinx health is important to inform future public health interventions and policy change;
- Our work highlights the significance of a CDoH approach to untangling the deleterious effects of SSB consumption on Latinx health.

**Public health implications—What are the key implications or messages for practitioners, policy makers and/or researchers in public health?**

- Our scoping review highlights that a CDoH lens can help inform future research beyond marketing exposure studies;
- Findings suggest that structural policy interventions and improved regulation of commercial practices are necessary to address higher exposure to marketing (and consumption) of SSBs among Latinx populations in the USA.

**Abstract:**

Commercial determinants of health (CDoHs) describe how corporate practices influence population health. This scoping review aimed to characterize the extant evidence base regarding how CDoH in the sugar-sweetened beverage (SSB) industry affects health and health-related outcomes among Latinx populations in the United States of America (USA). The present study was conducted in accordance with the JBI methodology for scoping reviews. Overall, 1236 references were identified and imported for screening. After duplicate removal, screening, and full-text eligibility assessment, 33 studies met all inclusion criteria. SSB marketing and advertising was the most frequently examined CDoH (61%), including advertising exposure, messaging strategies, and warning label interventions. SSB taxation studies projected reductions in consumption and obesity prevalence. Outcomes associated with health focused primarily on perceptions of marketing and purchasing intentions (94%). Additional studies examined the impact on knowledge, attitudes, beliefs, and behaviors (e.g., purchasing and consumption of SSBs) (66%), while a few studies included chronic disease (27%) or healthcare outcomes (6%). Evidence highlights several gaps in CDoH research associated with SSBs, with 94% of the included studies focused on understanding marketing exposure, signaling a need to examine other domains of CDoH, SSB industry practices, and impacts on health disparities. Findings suggest that structural policy interventions such as taxation and stronger regulation of commercial practices are necessary to address higher exposure to marketing and consumption of SSBs among Latinx populations in the USA.

## 1. Introduction

Commercial determinants of health (CDoHs) refer to “the systems, practices, and pathways through which commercial actors drive health and equity” [1]. Indeed, evidence shows that CDoH operates through multiple mechanisms (e.g., policy influence, marketing, production, and financial power) to shape population health outcomes [2]. Major multinational corporations are behind the production and promotion of unhealthy foods and beverages (such as sugar-sweetened beverages, SSBs), which have in turn been identified as key drivers of noncommunicable diseases (NCDs). SSBs (e.g., sodas, sweetened juices, energy drinks, and flavored teas) can deliver large quantities of sugar that can be rapidly absorbed and, in turn, increase caloric intake without feeling “full”, which can lead to a series of negative health outcomes, including (1) weight gain and obesity, (2) insulin resistance and type 2 diabetes, (3) cardiovascular disease (via higher levels of triglycerides, low-density lipoprotein [LDL], and high blood pressure), (4) nonalcoholic fat liver disease tied to higher fructose intake, and (5) dental issues linked to both SSBs acidity and sugar content [3,4,5,6].

In the United States of America (USA), the SSB market is largely under the control of three major corporations (the Coca Cola Company, PepsiCo, and Keurig Dr Pepper) with an estimated volume of sales of $315 billion in 2025 (projected to grow to $545 billion by 2032) [7]. According to the Centers for Disease Control and Prevention (CDCs), half of adults and two-thirds of children in the USA drank an SSB on any given day [8]. Studies have shown that SSB consumption among Latinos tends to be higher than the general population. Park and colleagues found that 58% of Latinx adults consumed SSBs two or more times a day and 36% did so three or more times [9], while Russo and colleagues estimated that Latinx children and adolescents consumed 14.1 and 18.4 fluid ounces of SSBs on a given day (a standard soda can in the USA contains 12 fluid ounces) [10]. Linked to SSB consumption, Latinx populations in the USA face significant health risks such as untreated tooth decay, obesity, and cardiovascular disease (CVD) [11]. Latinos depict higher rates of CVD risk factors such as obesity, hypertension, diabetes, and hyperlipidemia [12]. Hence, cardiovascular disease (e.g., coronary artery disease, angina, and myocardial infarction) is the leading cause of death among Latinos, with a prevalence rate of 8.2% [13]. CVD risk factors such as obesity and diabetes are more prevalent among Latinos compared to non-Latinx whites [14].

There is growing evidence that Latinos are disproportionately exposed to targeted commercial practices, especially in Spanish-language media, retail environments, and low-income neighborhoods [15]. This structural exposure contributes to dietary risk, information inequities, and barriers to healthy behaviors. Despite growing attention to CDoH globally [1,2,16] evidence specific to Latinos in the USA remains limited [15,17]. However, studies in Latin American countries such as Mexico and Colombia have found that the SSB industry uses several strategies to influence public policies at every stage of the policy cycle, undermining public health efforts to tackle obesity and non-communicable diseases in the region [17,18,19,20]. For instance, Ojeda and colleagues (2020) [18] studied the corporate political activity (CPA) strategies used by the SSB industry to undermine public health policymaking aimed at decreasing the consumption of SSB in Mexico. Their findings illustrate that the CPAs utilized by the SSB industry are similar to those used by the tobacco and alcohol industry, such as (1) lobbying, (2) actively promoting individual and parent’s responsibility for consumption behavior, (3) establishing relationships with policymakers, where former representatives of the industry work for the government and vice versa (“revolving doors”), (4) funding research studies to shape the evidence based on diet and public health, and (5) fragmenting and destabilization of the opposition by creating multiple voices against public health measures. As a result, the SSB industry’s use of CPA strategies influenced the formulation and implementation of public policies (e.g., SSB tax and front-of-package labeling) in Mexico, both directly and indirectly.

CDoH can be analyzed through seven interrelated domains that explain the set of activities corporate systems use to influence health outcomes and shape conditions in which priority populations live [1,16,21]. These domains of influence include: (1) political practices, (2) preference and perception shaping practices, (3) corporate social responsibility practices, (4) economic practices, (5) products and services, (6) employment practices, and (7) environmental practices. The way corporate activities can influence health outcomes is both indirect (distal domains) and direct (proximal domains). For example, when corporations make financial contributions to political parties (political practices), which influences government policy or processes in ways that are favorable to their commercial interests, this is indirectly shaping the environment in which priority populations live and work (proximal domain), utilize available products and services (proximal domain), and make decisions (e.g., to consume SSBs) that impact health and wellbeing. Within this typology, five priority populations are highlighted as being disproportionately impacted by CDoH. These include: (1) consumers, (2) workers, (3) disadvantaged groups, (4) vulnerable groups (e.g., pregnant women and children), and (5) local communities.

Existing research on CDoH among Latinx populations in the USA, one of the most historically disadvantaged populations in the country, is limited and tends to focus on marketing practices, particularly the differential exposure to unhealthy food, beverage, and alcohol advertising on Spanish-language media [22,23]. Hence, this scoping review leverages the CDoH framework to characterize the available literature examining the connections between SSB consumption and Latinx health and healthcare outcomes.

## 2. Materials and Methods

### 2.1. Protocol and Methodological Framework

The protocol for the present scoping review was published in the Open Science Framework portal [24], reported in accordance with the PRISMA extension for protocols (PRISMA-P), and conducted in accordance with the Joanna Briggs Institute (JBI) methodology for scoping reviews [25,26].

### 2.2. Research Questions and Eligibility Criteria

The research question was formulated following the JBI Population, Concept, and Context (PCC) framework. Eligible studies were required to be original empirical research involving individuals who identify as Hispanic/Latino/Latinx and reside in the USA. Studies including multiple racial/ethnic groups were considered eligible if findings specific to the Hispanic/Latino/Latinx subpopulation were clearly reported. Studies were included if they examined one or more commercial determinant of health (CDoH) practices, including political, scientific, marketing, supply chain/waste, labor, financial, or reputational domains, within the SSB sector.

Outcomes of interest included:

- Health outcomes: incidence or prevalence of non-communicable diseases (e.g., diabetes, obesity, cardiovascular disease, and cancer); knowledge, attitudes, beliefs and behaviors (e.g., consumption of SSBs);
- Healthcare outcomes: hospitalizations, healthcare utilization, or expenditures;
- Intermediate outcomes: marketing exposure rates, perceptions, purchase intentions, consumption of SSBs, media content, information environment, and policy outcomes.

Eligible study designs included quantitative, qualitative, and mixed-method empirical studies, as well as case studies. Only studies published in English from the year 2000 onwards were included. Studies conducted outside the USA or those not reporting Latinx-specific data were excluded. Editorials, commentaries, and opinion pieces were also excluded.

### 2.3. Search Strategy and Information Sources

The search strategy was developed by a librarian from the University of Massachusetts Amherst Libraries’ Evidence Synthesis Partnership (ESP) using the building block (i.e., sub-assembly) approach [27]. The following three conceptual elements were identified for the search: US Latinx populations (concept 1), SSBs (concept 2), and commercial determinants of health (concept 3). For each concept, a comprehensive set of controlled vocabulary and free-text search terms were compiled. Existing search filters were adapted for two of the concepts. The search block for US Latinx populations (concept 1) used the MLA Latinx Caucus search filter for Latinx/Hispanic US Population directly without modification [28]. Search terms used recently by the USDA Nutrition Evidence Synthesis Review team for SSBs (concept 2) were incorporated into the search strategy without retaining those terms pertaining to water, carbonated/sparkling water, milk/buttermilk/whey, and liquids [5]. Finally, search terms and controlled vocabulary for commercial determinants of health (concept 3) were identified from the conceptual framework developed by Gilmore et al. [1].

The draft search strategy was then compiled by ORing the set of terms for each concept and then ANDing the three concept blocks together. The draft search strategy was reviewed by the review team to provide an opportunity to include any missing terms and remove any terms included by mistake.

On 12 December 2025, the search strategy was executed in MEDLINE (Ovid), PsycInfo (EBSCOhost), Food Science Technology Abstracts (EBSCOhost), AGRICOLA (EBSCOhost), and Web of Science Core Collection (Clarivate Web of Sciences). Search results were limited by document type to remove editorials, commentaries, and opinion pieces. No limitations were placed on language of publication or publication date. Duplicate references were automatically removed upon upload to the Covidence platform (Melbourne, Australia; https://www.covidence.org/). Full documentation of the search strategy and translations are available as a Supplemental Material (Tables S1–S5).

### 2.4. Screening Process

All identified records were imported into Covidence for screening. Title and abstract screening were conducted independently by five reviewers (MPL, MPA, ER, JM, and IC) to exclude studies that did not meet the eligibility criteria. All stages of the screening process (title/abstract and full-text review) were conducted in duplicate, with each record independently assessed by two reviewers to ensure methodological rigor. Discrepancies were resolved through a third reviewer (DLC). Studies deemed potentially eligible were retrieved for full-text review, which was conducted independently by six reviewers, following the same duplicate screening process. Consistent with the objectives of a scoping review, no formal assessment of risk of bias or methodological quality was conducted.

### 2.5. Data Extraction, Synthesis, and Analysis

Data were extracted from all included studies using standardized forms. Extracted variables included: study characteristics, population, setting, sector, outcome type, study design, context, funding sources/conflicts of interest, and recommendations. Four reviewers (MPA, ER, JM, and IC) independently conducted data extraction, and all articles were reviewed by a second reviewer to ensure accuracy and consistency. The findings were synthesized into the following three tables: (1) study characteristics, (2) commercial determinants of health, and (3) study recommendations. Additionally, a narrative synthesis was conducted to summarize the main findings.

## 3. Results

**Figure 1:**
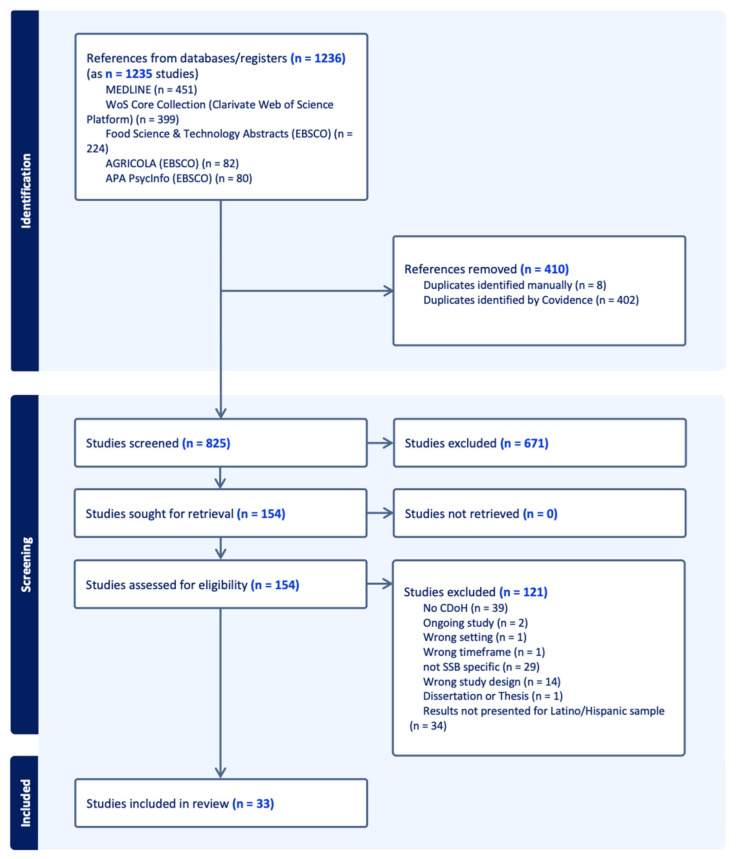
PRISMA flow diagram of systematic review literature selection.

A total of 1236 references were imported for screening (representing 1235 studies). Eight duplicates were identified manually, and 402 duplicates were identified and removed using Covidence. After duplicate removal, 825 studies were screened by title and abstract, of which 671 were excluded as irrelevant. A total of 154 full-text articles were assessed for eligibility. Of these, 121 studies were excluded for the following reasons: not addressing commercial determinants of health (*n* = 39); results not presented for Latinx samples (*n* = 34); not specific to SSBs (*n* = 29); wrong study design (*n* = 14); ongoing study (*n* = 2); dissertation or thesis (*n* = 1); wrong setting (*n* = 1); and wrong timeframe (*n* = 1). No studies were ongoing or awaiting classification. In total, 33 studies met the inclusion criteria and were included in the review. The PRISMA flow diagram is shown in Figure 1.

A summary of the type, CDoH, and location of the studies included in the sample is provided in Table 1. Across 33 included studies published between 2007 and 2025, evidence increased substantially from 2020 onwards. The peak year was 2022 (*n* = 6; 18.2%) [29,30,31,32,33,34]. This was followed by 2021 (*n* = 5; 15.2%) [35,36,37,38,39] and 2020 (*n* = 4; 12.1%) [40,41,42,43]. Recent years remained productive, including 2023 to 2025 (*n* = 6; 18.1%) [44,45,46,47,48,49], whereas 2007–2019 contributed only a few studies [50,51,52,53,54,55,56,57,58,59,60,61]. Most studies were observational and non-randomized (*n* = 17; 51.5%), typically cross-sectional surveys, audits, or secondary analyses [32,33,34,38,43,45,49,50,51,52,54,55,56,57,60,61]. Randomized designs were also used (*n* = 7; 21.2%), including online and field-based RCTs testing warning labels, message framing, text messages, and counter marketing strategies [31,36,37,39,44,46,59]. Interestingly, policy modeling via microsimulations accounted for five studies (15.2%) [30,41,48,53,58]. A smaller subset used qualitative methods [29,40,42], and only one utilized a longitudinal cohort design [47].

When grouped by core objectives, studies most frequently examined marketing and communication mechanisms, including advertising exposure, warning labels, message framing, counter marketing, and social media engagement [29,31,33,34,35,36,37,39,40,43,44,46,50,51,54,56,59]. The second cluster focused on fiscal policy, including taxation and minimum price laws, either through real-world evaluations or simulation modeling [30,38,41,47,48,53]. Another group assessed neighborhood and retail environments and structural inequities [32,45,49,55,57,60]. Finally, other information-environment objectives were uncommon [52,61].

Consistent with the objective pattern, the most frequent commercial determinant category was marketing and advertising of SSBs [29,36,39,44,46,50,51,59]. Taxation of SSBs and neighborhood-level inequities were each represented in six studies [30,32,38,41,45,47,48,49,53,55,57,60]. Other CDoH domains were rare [52,61]. In terms of place in the USA, over one-third of the evidence came from national-scale samples or analyses [34,37,41,43,44,50,51,53,54,58]. California was the most common setting [31,33,46,48,57,60] followed by New York City [30,39]. Most studies were conducted in urban settings [29,30,31,32,33,38,39,46,47,56,57,59]. Only one study was explicitly rural (South Texas colonias [52]). Urbanicity was not reported in several modeling or national-level studies [37,41,48,53,54].

### 3.1. Marketing and Advertising of SSBs

Twenty of the 33 included studies (60.6%) examined marketing exposure, advertising practices, messaging strategies, purchase intentions, or warning label policies related to SSBs [29,30,33,34,35,36,37,39,40,43,44,46,50,51,53,56,58,59]. Outcomes most frequently assessed included perceptions of marketing messages, purchase intentions, advertising exposure rates, and responses to warning label policies. Only one study projected long-term obesity outcomes [58], and none measured hospitalization or healthcare utilization.

Experimental and messaging-based studies evaluated warning labels, communication strategies, and counter marketing approaches. These studies assessed perceived effectiveness, perceived harms, and intentions to select or purchase SSBs. Empowerment-based messaging was perceived as more effective and generated less reactance than fear-based messaging, while counter marketing strategies reduced misperceptions of product healthfulness and purchase intentions [31,36,37,44,46].

Advertising exposure disparities by race and ethnicity were documented across studies. Higher exposure to unhealthy beverage advertising was observed in Spanish-language media and in neighborhoods with higher Latinx composition. Latinx youth reported frequent exposure to SSB advertising, and higher engagement with food and beverage brands was observed among less-acculturated adolescents [29,33,43,50,54].

Retail and environmental marketing practices were also assessed. Studies reported high exposure to price promotions, social media marketing, and environmental advertising, along with perceptions that SSBs were more accessible than water. Product placement, pricing, and availability across stores, schools, and public settings were associated with purchasing behaviors and exposure patterns [34,39,40,42,51,56,59]. Across marketing-related studies, outcomes were primarily limited to perceptions, intentions, and exposure measures. Direct clinical outcomes were rare [58], and no studies assessed hospitalization outcomes.

### 3.2. Taxation of Sugar-Sweetened Beverages

Six studies evaluated SSB taxation policies, including excise taxes, minimum price laws, and alternative tax designs. These studies assessed projected or observed changes in SSB consumption, BMI, obesity prevalence, cardiometabolic outcomes, and economic impacts [30,38,41,47,48,53]. Modeling studies projected reductions in SSB consumption and obesity prevalence following taxation, with some studies showing larger relative effects among Latinx populations. Additional projections included impacts on cardiovascular disease, diabetes, healthcare costs, and government revenue [30,41,48,53].

Empirical evaluations reported mixed findings. Some studies identified associations between taxation and changes in BMI or purchasing behaviors, while others reported no statistically significant differences. Adolescents were more price-responsive than adults [38,47].

### 3.3. Neighborhood-Level Inequities

Six studies examined neighborhood environments and structural inequities related to SSB exposure and consumption [32,45,49,55,57,60]. These studies assessed high density of soda outlets in low-income environments, product availability, purchasing patterns, pricing differences, and associations with sociodemographic characteristics. Higher SSB consumption and purchasing were observed in areas with high density of unhealthy food outlets and among populations in lower-income or higher Latinx composition neighborhoods. Pricing disparities were also documented, with SSBs often costing less than healthier alternatives such as milk [32,45,49,55,57,60]. Some studies assessed associations with BMI and obesity outcomes [45,60], although findings were mixed and not consistently associated with environmental measures.

#### Other/Information Environment

Two studies were categorized under other information environments and examined structural contributors to beverage access [52,61]. These studies assessed food outlet density, proximity to convenience stores, and characteristics of mobile and home-based food vendors [52,61]. Neither study reported healthcare outcomes, hospitalization, or obesity projections.

### 3.4. Results by Policy and Action Recommendation

Across the 33 articles included in this scoping review, future action recommendations clustered around the following five policy domains: SSB taxation, warning labels, availability restrictions, advertising regulation, and education interventions. These approaches aim to advance health equity among Latinx, low-income, and minority youth populations. SSB taxation emerged as the most frequently recommended policy lever. Authors consistently identified excise taxes as an evidence-based strategy to reduce purchases and lower obesity risk among children and adolescents [31,34,38,42,47,48,53,57]. Importantly, two studies emphasized that taxation should be designed with equity in mind [48,53]. For example, tiered or sugar-content-based taxes were suggested as potentially more effective than volume-based taxes, as they can target high-sugar products more precisely [41]. Relative beverage pricing with making healthier alternatives such as water and milk more affordable was emphasized, as SSBs have historically been cheaper in many low-income communities [55].

For SSB availability, limiting SSB access in schools [51] and modifying immediate food environments [40,61], including convenience stores [32] and checkout areas [49], was recommended, where high-exposure marketing disproportionately affects youth. Interventions targeting “food desert” retailers [52] and neighborhood inequities were framed as critical for improving nutrition equity in low-SES and minority communities [31]. These environmental changes were positioned as structural solutions that reduce reliance on individual behavior change alone [32,40,45].

Regarding warning labels, researchers advocated for prominent front-of-package disclosures to increase transparency about sugar content and health risks [31,34,59]. Two studies emphasized that including visual images, rather than text-only labels, may improve accessibility for individuals with low English proficiency [36,39], including unacculturated Latinx populations [44]. This approach would enhance informational equity for more informed decision-making.

Advertising regulation was also strongly emphasized, particularly in response to racially targeted marketing practices. Studies urged policymakers to address disproportionate exposure to SSB advertising among minority and low-SES youth in schools [51] and through television and social media [34,35,50]. Bilingual advertisements in Spanish and English were also noted as targeted marketing towards Latinx youth, further underscoring the need to monitor across diverse media platforms [29,50]. More research is needed to determine to what extent overall SSB advertising exposure is linked to actual behaviors [54].

Education- and community-based interventions were recommended to complement structural policy approaches [31]. Tailored public service announcements [42,60] and empowerment-based communication strategies [46] were proposed to address individual-level barriers and promote collective agency within marginalized communities. Instead of education as a standalone solution, authors generally framed it as a supportive strategy to strengthen the impact of broader regulatory policies [60].

A limited number of studies incorporated empowerment-based strategies, emphasizing community agency to counter industry marketing [46]. Similarly, only a few examined the use of digital data and social media analytics to understand exposure and engagement with food and beverage marketing [35]. One study uniquely focused on informal or home-based food environments, highlighting the role of small-scale vendors in shaping access to food [52]. Other less common approaches included financial incentives combined with labeling interventions [59] and the use of mobile health (mHealth) strategies to influence behavior [39].

Table 2 provides a heatmap of topic areas and outcomes of interest for all 33 studies. In it, we show CDoH clustered around the following four topic areas: (1) marketing/advertising of SSBs, (2) taxation of SSBs, (3) neighborhood level inequities, and (4) other/information environment; and four outcomes of interest: (1) health outcomes (e.g., diabetes, obesity, CVD); (2) knowledge/attitudes/behavior (intentions, perceptions, purchasing, and consumption); (3) healthcare outcomes (hospitalization, healthcare utilization, or expenditures), and (4) marketing exposure rates, purchase intentions and policy outcomes. Each box depicts the number of articles that covered the intersection of topics and outcomes and is colored from red—the most articles—to gray—the least (e.g., most articles [*n* = 19] focused on SSB marketing/advertising [topic area] and measured marketing exposure rates as their outcome of interest).

## 4. Discussion

This scoping review is the first, to our knowledge, to systematically characterize the existing evidence on the connections between SSB consumption and Latinx health and healthcare outcomes through a CDoH lens within the USA. Overall, findings highlight that research in this field remains limited in scope and concentrated primarily on marketing and communication practices, with significant literature gaps regarding structural, political, and economic domains of SBB corporate influence and its consequences on the health and wellbeing of Latinx populations in the USA.

The evidence base consistently indicates that Latinos are disproportionately exposed to targeted marketing practices, particularly through Spanish-language media and retail environments, which may contribute to health inequities [23]. However, the literature remains fragmented, with limited exploration of health and health care outcomes and insufficient integration of culturally grounded and intersectional perspectives. In our review, studies predominantly focused on marketing and advertising of SSBs, signaling a focus on more proximal determinants (e.g., messaging, exposure, and consumer behavior) rather than more distal (structural) determinants (e.g., corporate practices, policy influence). Moreover, most studies in the present review assessed perceptions, intentions, and consumption of SSBs, rather than chronic disease status or healthcare outcomes, which is like other studies focused on “measurable” (short-term) behavioral outcomes over (long-term) health impacts [1,16,21]. Indeed, few studies examined corporate influence on policy or structural conditions, highlighting a critical gap in understanding how power dynamics and governance shape health inequities in Latinx populations in the USA [62,63].

Importantly, findings reinforce the role of targeted marketing as a key mechanism through which corporate practices may shape health inequities. Higher exposure to SSB advertising among Latinx populations, particularly youth and less acculturated individuals, is consistent with previous research demonstrating that minority populations are disproportionately targeted by unhealthy commodity industries [8,9]. These patterns suggest that commercial practices are strategically designed to appeal to sociocultural values. Indeed, culturally grounded frameworks were only addressed in very few studies [27,37]. Furthermore, although heterogeneity across populations was occasionally explored, only a subset of studies explicitly examined within-group differences [14,41], indicating an underdeveloped focus on intersectionality within the extant literature. This is particularly important given the diversity within Latinx populations, where factors such as immigration status, socioeconomic position, and generational differences may shape SSB exposure/preference, consumption and related health outcomes.

Across the included studies, recommendations clustered around the following five main domains: taxation, warning labels, availability restrictions, advertising regulation, and education interventions. Among these, SSB taxation emerged as the most consistently supported strategy, aligning with evidence (in Latin American and elsewhere) demonstrating its effectiveness in reducing consumption and improving population health outcomes [30,41,48,53]. The emphasis on equity-oriented tax design, such as sugar-content-based taxation, seems particularly promising. Warning labels and advertising regulations were also strongly endorsed, especially in response to documented disparities in marketing exposure, with evidence suggesting that visual and bilingual labels may improve accessibility for Latinx populations [34,35,36]. Environmental interventions, including restrictions on SSB availability in schools and retail settings, were framed as structural approaches that reduce reliance on individual behavior change. While education-based interventions were frequently recommended, their effectiveness appears limited when implemented in isolation, reinforcing the need for integration with broader regulatory strategies.

Based on the findings of this review, future research and policy efforts should adopt a more comprehensive and equity-oriented approach to addressing the impacts of SSB consumption among Latinx populations in the USA using a CDoH approach. Specifically, there is a need to: (1) expand research beyond marketing to include political and economic determinants; (2) incorporate culturally grounded and intersectional frameworks; and (3) prioritize the evaluation of long-term health outcomes. From a policy perspective, coordinated structural efforts (e.g., combining taxation, regulation of marketing and availability, with culturally responsive approaches) may be more effective. Here, we can learn more robust approaches taken across Latin America to tackle corporate influence over fiscal and regulatory policy [62,63]. A recent study shows, for instance, that in Mexico food and beverage corporate influence was reduced in part because of nutrition experts being embedded within government policymaking. Conversely, in Brazil, industry’s power has endured due to presidential preference towards government–industry partnership to implement its popular antihunger program [64]. This resembles current practices within the USA, where there is the influence of the Coca Cola Company on the Centers for Disease Control and Prevention to move away from SSB taxation, providing an opportunity for research to examine CDoH influence on federal policy [65]. Overall, the findings of this scoping review and the literature examining CDoH influence of SSB industry internationally suggest that reducing SSB consumption among Latinos will require both, regulating harmful corporate practices and transforming the structural economic and policy conditions.

### Limitations

This scoping review has several limitations that should be considered when interpreting the findings. First, no formal assessment of risk of bias or methodological quality was conducted; however, this approach is consistent with the purpose of scoping reviews, which aim to provide a comprehensive mapping of the available evidence rather than evaluate study quality, allowing for the inclusion of a broad range of study designs [66]. Second, the review was limited to studies published in English. Third, although a comprehensive search strategy was implemented, it is possible that some relevant studies, particularly the gray literature or unpublished reports, were not identified. Nevertheless, the search strategy was developed by an experienced academic librarian (ET) and conducted across multiple major databases, which strengthens the rigor and reproducibility of the review. Finally, the heterogeneity of study designs, outcomes, and measures limited comparability across studies and precluded quantitative synthesis. Additionally, most studies focused on behavioral outcomes (e.g., observational or perception-based studies) rather than longer-term health or healthcare outcomes, which restricts the ability to draw conclusions about the CDoH dimensions of SSB consumption on Latinx health.

## 5. Conclusions

The evidence analyzed in this scoping review highlights the gap in research on health outcomes associated with CDoH and SSBs, with most of the included studies focused on marketing/advertising issues. Hence, findings suggest that structural policy interventions (e.g., restrictions on SSB marketing, taxation policies, and warning labels) and improved regulation of commercial practices (e.g., regulating SSB marketing reach and content), while key to address higher exposure to marketing of SSBs among Latinx populations in the USA, remain a significant gap in the extant literature.

## Figures and Tables

**Table 1:** Summary of studies included in the scoping review by type of study, CDoH and share of Latinx participants in each study.

Author(s) (Year)	Type of Study	CDoH	Location
Abdul Razak et al. (2025) [45]	Quantitative, secondary analysis	Neighborhood Level Inequities	Massachusetts and New Hampshire
Wolf et al. (2025) [44]	Online RCT	Marketing/Advertising of SSBs	National
Zhou et al. (2025) [46]	RCT	Marketing/Advertising of SSBs	Merced, California
Jones-Smith et al. (2024) [47]	Longitudinal cohort	Taxation of SSBs	Seattle
Lee et al. (2024) [48]	Microsimulation	Taxation of SSBs	California
Marinello et al. (2023) [49]	Cross-sectional	Neighborhood Level Inequities	Northern California
DuPont-Reyes et al. (2022) [29]	Quantitative content analysis	Marketing/Advertising of SSBs	Houston, Texas
Grummon & Golden (2022) [30]	Microsimulation	Taxation of SSBs	New York City, New York
Grummon et al. (2022) [31]	Randomized experiment	Marketing/Advertising of SSBs	San Francisco, California
Winkler et al. (2022) [32]	Observational	Neighborhood Level Inequities	Minneapolis–St. Paul, Minnesota
Zahid et al. (2022) [33]	Cross-sectional	Marketing/Advertising of SSBs	San Francisco/Oakland, California
Choi et al. (2022) [34]	Quantitative observational	Marketing/Advertising of SSBs	National
Rummo et al. (2021) [35]	Quantitative	Marketing/Advertising of SSBs	National
Hall et al. (2021) [36]	Randomized experiment	Marketing/Advertising of SSBs	National
Woo Baidal et al. (2021) [39]	RCT	Marketing/Advertising of SSBs	New York City, New York
Krieger et al. (2021) [37]	RCT	Marketing/Advertising of SSBs	National
Edmondson et al. (2021) [38]	Quantitative survey	Taxation of SSBs	Philadelphia, Pennsylvania
Castellanos & Miller (2020) [40]	Qualitative, focus groups	Marketing/Advertising of SSBs	Southwest Ohio
Fleming-Milici & Harris (2020) [43]	Quantitative survey	Marketing/Advertising of SSBs	National
Lee et al. (2020) [41]	Microsimulation	Taxation of SSBs	National
Khandelwal & Salazar (2020) [42]	Qualitative	Marketing/Advertising of SSBs	Canyon, Texas
Zahid et al. (2022) [33]	Cross-sectional	Neighborhood Level Inequities	Four Bay Area cities, California
Grummon et al. (2019) [58]	Microsimulation	Marketing/Advertising of SSBs	National
Franckle et al. (2018) [59]	RCT	Marketing/Advertising of SSBs	Chelsea, Massachusetts
Wong et al. (2018) [60]	Cross-sectional	Neighborhood Level Inequities	California
Lucan et al. (2017) [56]	Cross-sectional	Marketing/Advertising of SSBs	The Bronx, New York
Kern et al. (2016) [55]	Cross-sectional	Neighborhood Level Inequities	Forty-one states
Kumar et al. (2015) [54]	Cross-sectional	Marketing/Advertising of SSBs	National
Kristensen et al. (2014) [53]	Microsimulation	Taxation of SSBs	National
Fleming-Milici et al. (2013) [50]	Quantitative exposure study	Marketing/Advertising of SSBs	National
Richmond et al. (2013) [61]	Quantitative	Other/Information Environment	Massachusetts
Valdez et al. (2012) [52]	Quantitative	Other/Information Environment	South Texas
Johnston et al. (2007) [51]	Cross-sectional survey	Marketing/Advertising of SSBs	National

**Table 2:** Heatmap of the evidence based on topic areas and outcomes of interest.

Topic Area	Outcome of Interest			
	Health Outcomes (e.g., Diabetes, Obesity, CVD)	Knowledge/Attitudes/Behavior (Intentions, Perceptions, Purchasing, and Consumption)	Healthcare Outcomes (Hospitalization, Healthcare Utilization, or Expenditures)	Marketing Exposure Rates, Purchase Intentions and Policy Outcomes
Marketing/Advertising of SSBs	3	12	0	19
Taxation of SSBs	5	5	2	5
Neighborhood Level Inequities	1	4	0	6
Other/Information Environment	0	1	0	1

Note: Each box depicts the number of articles that covered the intersection of topics and outcomes and is colored from red—the most articles—to gray—the least (e.g., most articles [*n* = 19] focused on SSB marketing/advertising [topic area] and measured marketing exposure rates as their outcome of interest).

## Data Availability

No new data were created or analyzed in this study.

## Supplementary Materials

The following supporting information can be downloaded at https://www.mdpi.com/article/10.3390/ijerph23060766/s1, Table S1: Search Strategy and Translations (Medline All); Table S2: Search Strategy and Translations (PsychInfo); Table S3: Search Strategy and Translations (Food Science Technology Abstracts); Table S4: Search Strategy and Translations (AGRICOLA); Table S5: Search Strategy and Translations (AGRICOLA # 2); Table S6: Preferred Reporting Items for Systematic reviews and Meta-Analyses extension for Scoping Reviews (PRISMA-ScR) Checklist. Ref. [67] was cited in Supplementary Materials.

## Abbreviations

The following abbreviations are used in this manuscript:

BMI	Body Mass Index
CDCs	Centers for Disease Control and Prevention
CDoHs	Commercial Determinants of Health
ESP	Evidence Synthesis Partnership
SSBs	Sugar-Sweetened Beverages
USA	United States of America
