# Supplementary material for: Commercial Determinants of Latinx Health: A Scoping Review of Sugar-Sweetened Beverages in the USA
Source: Int J Environ Res Public Health. 2026 Jun 6;23(6):766. doi: 10.3390/ijerph23060766 (PMC13299247; doi:10.3390/ijerph23060766)
Supplement: Supplementary file 1 [file ijerph-23-00766-s001.zip › ijerph-4348714-supplementary.pdf]

Supplemental Material

Table S1. Search strategy & translations

Database: MEDLINE All

Platform: Ovid

Temporal Coverage: Database inception to December 12, 2025

#	ID	Explanatory Note	Search Terms
1	1a	MLA search hedge: Latinx/Hispanic US Population 1	exp "Hispanic or Latino"/ or Hispanic*.ab,ti. or hispano*.ab,ti. or hispana*.ab,ti. or Latino*.ab,ti. or Latina*.ab,ti. or Latine*.ab,ti. or Latinu*.ab,ti. or Latinx.ab,ti. or "Spanish American*".ab,ti. or "Mexican American*".ab,ti. or Chicana*.ab,ti. or Chicano*.ab,ti. or Chicanx.ab,ti. or exp "Puerto Rico"/ or "Puerto Rico".ab,ti. or "Puerto Rican*".ab,ti. or Boricua*.ab,ti. or "Cuban American*".ab,ti.
2	1b	MLA search hedge: Latinx/Hispanic US Population 2	"Spanish speak*".ab,ti. or Argentina/ or Argentina.ab,ti. or Argentine*.ab,ti. or Argentinian*.ab,ti. or Bolivia/ or Bolivia.ab,ti. or Bolivian*.ab,ti. or Brazil/ or Brazil.ab,ti. or Brazilian*.ab,ti. or Chile/ or Chile.ab,ti. or Chilean*.ab,ti. or Colombia/ or Colombia.ab,ti. or Colombian*.ab,ti. or "Costa Rica"/ or "Costa Rica".ab,ti. or "Costa Rican*".ab,ti. or Cuba/ or Cuba.ab,ti. or Cuban*.ab,ti. or Hispaniola.ab,ti. or "Dominican Republic"/ or Dominican*.ab,ti. or Ecuador/ or Ecuador.ab,ti. or Ecuadorian*.ab,ti. or Ecuadorean*.ab,ti. or "El Salvador"/ or "El Salvador".ab,ti. or Salvadorean*.ab,ti. or Salvadoran*.ab,ti. or Salvadorian*.ab,ti. or Guatemala/ or Guatemala.ab,ti. or Guatemalan*.ab,ti. or Honduras/ or Honduras.ab,ti. or Honduran*.ab,ti. or Mexico/ or Mexico.ab,ti. or Mexican*.ab,ti. or Nicaragua/ or Nicaragua.ab,ti. or Nicaraguan*.ab,ti. or Panama/ or Panama.ab,ti. or Panamanian*.ab,ti. or Paraguay/ or Paraguay.ab,ti. or Paraguayan*.ab,ti. or Peru/ or Peru.ab,ti. or Peruvian*.ab,ti. or Uruguay/ or Uruguay.ab,ti. or Uruguayan*.ab,ti. or Venezuela/ or Venezuela.ab,ti. or Venezuelan*.ab,ti. or "Central America*".ab,ti. or "Central America"/ or Caribbean*.ab,ti. or "Caribbean Region"/ or "South America"/ or "South America*".ab,ti. or "Latin America*".ab,ti. or "Latin America"/
3	1c	MLA search hedge: Latinx/Hispanic US Population 3	exp "united states"/ or "united state*".ab,ti. or USA.ab,ti. or "north america"/ or Appalachia*.ab,ti. or "great lakes".ab,ti. or "mid atlantic state*".ab,ti. or "mid atlantic region*".ab,ti. or "middle atlantic state*".ab,ti. or "middle atlantic region*".ab,ti. or "midwestern us".ab,ti. or "midwestern state*".ab,ti. or "midwest state*".ab,ti. or "midwest us".ab,ti. or "great plains".ab,ti. or heartland.ab,ti. or "new england".ab,ti. or "northeastern us".ab,ti. or "northeastern state*".ab,ti. or "northeast state*".ab,ti. or "northeast us".ab,ti. or "pacific northwest".ab,ti. or "northwestern us".ab,ti. or "northwest us".ab,ti. or "northwestern state*".ab,ti. or "northwest state*".ab,ti. or "pacific state*".ab,ti. or "southeast state*".ab,ti. or "southeastern state*".ab,ti. or "southeast us".ab,ti. or "southeastern us".ab,ti. or "southern state*".ab,ti. or "southern us".ab,ti. or "southwest state*".ab,ti. or "southwestern state*".ab,ti. or "southwest us".ab,ti. or "southwestern us".ab,ti. or "deep south".ab,ti. or "black belt".ab,ti. or "rust belt".ab,ti. or "district of Columbia".ab,ti. or "Washington dc".ab,ti. or Alabama.ab,ti. or Alaska.ab,ti. or Arizona.ab,ti. or Arkansas.ab,ti. or California.ab,ti. or Colorado.ab,ti. or Connecticut.ab,ti. or Delaware.ab,ti. or Florida.ab,ti. or Georgia.ab,ti. or Hawaii.ab,ti. or "Hawai i".ab,ti. or Idaho.ab,ti. or Illinois.ab,ti. or Indiana.ab,ti. or Iowa.ab,ti. or Kansas.ab,ti. or Kentucky.ab,ti. or Louisiana.ab,ti. or Maine.ab,ti. or Maryland.ab,ti. or Massachusetts.ab,ti. or Michigan.ab,ti. or Minnesota.ab,ti. or Mississippi.ab,ti. or Missouri.ab,ti. or Montana.ab,ti. or Nebraska.ab,ti. or Nevada.ab,ti. or "New Hampshire".ab,ti. or "New Jersey".ab,ti. or "New Mexico".ab,ti. or "New York".ab,ti. or "North Carolina".ab,ti. or "North Dakota".ab,ti. or Ohio.ab,ti. or Oklahoma.ab,ti. or Oregon.ab,ti. or Pennsylvania.ab,ti. or "Rhode Island".ab,ti. or "South Carolina".ab,ti. or "South Dakota".ab,ti. or Tennessee.ab,ti. or Texas.ab,ti. or Utah.ab,ti. or Vermont.ab,ti. or Virginia.ab,ti. or Washington.ab,ti. or "West Virginia".ab,ti. or Wisconsin.ab,ti. or Wyoming.ab,ti. or America*.ab,ti. or Americas/ or "Western Hemisphere".ab,ti.
4	1bc	MLA search hedge: Latinx/Hispanic US Population 2&3	2 and 3
5	1a+bc	MLA search hedge: Latinx/Hispanic US Population	1 or 4
6	2cv	CDoH: controlled vocabulary	exp Taxes/ OR exp Marketing/ OR Social Media/ OR exp Food Industry/
7	2ft	CDoH: free text	"marketing".ti,ab,kf. OR advertis*.ti,ab,kf. OR "price promoti*".ti,ab,kf. OR "food environment*".ti,ab,kf. OR "food desert?".ti,ab,kf. OR corporation?.ti,ab,kf. OR "corporate social responsibility".ti,ab,kf. OR (corporat* and CSR).ti,ab,kf. OR

			lobbying.ti,ab,kf. OR "social media".ti,ab,kf. OR (facebook or twitter or tweet* or instagram or snapchat or youtube or tiktok or "viral content" or meme?).ti,ab,kf. OR (online and influencer?).ti,ab,kf. OR "working condition".ti,ab,kf. OR ((labor or work* or industr*) and union?).ti,ab,kf. OR monopol*.ti,ab,kf. OR (oligopoly or oligopolies).ti,ab,kf. OR philanthrop*.ti,ab,kf. OR capitalis*.ti,ab,kf OR neoliberal*.ti,ab,kf OR "political economy".ti,ab,kf OR "market concentration".ti,ab,kf OR "civil society capture".ti,ab,kf OR "think tank*".ti,ab,kf OR "grocery industry".ti,ab,kf
8	2px	CDoH: proximity operator terms	(commercial adj3 determinant?).ti,ab,kf. OR (industr* adj6 influenc*).ti,ab,kf. OR (digital* adj3 engage*).ti,ab,kf. OR (suppress* adj2 wage?).ti,ab,kf. OR (price? adj3 manipulat*).ti,ab,kf. OR (tax* adj6 (avoid* or credit* or dodg* or strateg*)).ti,ab,kf. OR (brand* adj3 (environment* or social or green*)).ti,ab,kf. OR (food adj4 retail*).ti,ab,kf
9	2combined	CDoH: combined	or/6-8
10	3cv	Sugar-sweetened beverages (USDA NESR): controlled vocabulary	Beverages/ OR Carbonated Beverages/ OR Energy Drinks/ OR "Fruit and Vegetable Juices"/ OR Coffee/ OR Tea/
11	3ft	Sugar-sweetened beverages (USDA NESR): free text	beverage?.ti,ab,kf. OR "caloric drink*".ti,ab,kf. OR "sport? drink?".ti,ab,kf. OR "protein drink?".ti,ab,kf. OR "fortified drink?".ti,ab,kf. OR "sweetened drink?".ti,ab,kf. OR "sweet drink?".ti,ab,kf. OR "sugary drink?".ti,ab,kf. OR "dairy drink?".ti,ab,kf. OR "chocolate drink?".ti,ab,kf. OR "nutritional drink?".ti,ab,kf. OR smoothie?.ti,ab,kf. OR "protein shake?".ti,ab,kf. OR "meal replacement?".ti,ab,kf. OR "carbonated drink?".ti,ab,kf. OR "soft drink?".ti,ab,kf. OR soda?.ti,ab,kf. OR "caffeinated drink?".ti,ab,kf. OR "energy drink?".ti,ab,kf. OR "sugar sweetened drink?".ti,ab,kf. OR juice?.ti,ab,kf. OR "fruit drink?".ti,ab,kf. OR "fizzy drink?".ti,ab,kf. OR coffee?.ti,ab,kf. OR tea.ti,ab,kf. OR ((drink? or beverage? or diet*) and SSB?).ti,ab,kf.
12	3combined	Sugar-sweetened beverages (USDA NESR): combined	or/10-11
13		Complete search strategy	and/5,9,12

Table S2. Search strategy & translations

Database: PsycInfo

Platform: EBSCOhost

Temporal Coverage: Database inception to December 12, 2025

#	ID	Explanatory Note	Search Terms
1	1	MLA search hedge: Latinx/Hispanic US Population	(((DE "Latinos/Latinas" OR DE "Mexican Americans") OR (TI Hispanic* OR AB Hispanic*) OR (TI hispano* OR AB hispano*) OR (TI hispana* OR AB hispana) OR (TI Hispaniola OR AB Hispaniola) OR (TI Latino* OR AB Latino*) OR (TI Latina* OR AB Latina*) OR (TI Latine* OR AB Latine*) OR (TI Latinu* OR AB Latinu*) OR (TI Latinx OR AB Latinx) OR (TI "Spanish American*" OR AB "Spanish American*") OR (TI "Mexican American*" OR AB "Mexican American*") OR (TI Chicana* OR AB Chicana*) OR (TI Chicano* OR AB Chicano*) OR (TI Chicanx OR AB Chicanx) OR (TI "Puerto Rico" OR AB "Puerto Rico") OR (TI "Puerto Rican*" OR AB "Puerto Rican*") OR (TI Boricua* OR AB Boricua*) OR (TI "Cuban American*" OR AB "Cuban American*")) OR (((TI "Spanish speak*" OR AB "Spanish speak*") OR (TI Argentina OR AB Argentina) OR (TI Argentine* OR AB Argentine*) OR (TI Argentinian* OR AB Argentinian*) OR (TI Bolivia OR AB Bolivia) OR (TI Bolivian* OR AB Bolivian*) OR (TI Brazil OR AB Brazil) OR (TI Brazilian* OR AB Brazilian*) OR (TI Chile OR AB Chile) OR (TI Chilean* OR AB Chilean*) OR (TI Colombia OR AB Colombia) OR (TI Colombian* OR AB Colombian*) OR (TI "Costa Rica" OR AB "Costa Rica") OR (TI "Costa Rican*" OR AB "Costa Rican*") OR (TI Cub OR AB Cuba) OR (TI Cuban* OR AB Cuban*) OR (TI Dominican* OR AB Dominican*) OR (TI Ecuador OR AB Ecuador) OR (TI Ecuadorian* OR AB Ecuadorian*) OR (TI Ecuadorean* OR AB Ecuadorean*) OR (TI "El Salvador" OR AB "El Salvador") OR (TI Salvadorean* OR AB Salvadorean*) OR (TI Salvadoran* OR AB Salvadoran*) OR (TI Salvadorian* OR AB Salvadorian*) OR (TI Guatemala OR AB Guatemala) OR (TI Guatemalan* OR AB Guatemalan*) OR (TI Honduras OR AB Honduras) OR (TI Honduran* OR AB Honduran*) OR (TI Mexico OR AB Mexico) OR (TI Mexican* OR AB Mexican*) OR (TI Nicaragua OR AB Nicaragua) OR (TI Nicaraguan* OR AB Nicaraguan*) OR (TI Panama OR AB Panama) OR (TI Panamanian* OR AB Panamanian*) OR (TI Paraguay OR AB Paraguay) OR (TI Paraguayan* OR AB Paraguayan*) OR (TI Peru OR AB Peru) OR (TI Peruvian* OR AB Peruvian*) OR (TI Uruguay OR AB Uruguay) OR (TI Uruguayan* OR AB Uruguayan*) OR (TI Venezuela OR AB Venezuela) OR (TI Venezuelan* OR AB Venezuelan*) OR (TI "Central America*" OR AB "Central America*") OR (TI "Spanish Caribbean*" OR AB "Spanish Caribbean*") OR (TI "South America*" OR AB "South America*") OR (TI "Latin America*" OR AB "Latin America*")) AND ((TI "united state*" OR AB "united state*") OR (TI USA OR AB USA) OR (TI Appalachia* OR AB Appalachia*) OR (TI "great lakes" OR AB "great lakes") OR (TI "mid atlantic state*" OR AB "mid atlantic state*") OR (TI "mid atlantic region*" OR AB "mid atlantic region*") OR (TI "middle atlantic state*" OR AB "middle atlantic state*") OR (TI "middle atlantic region*" OR AB "middle atlantic region*") OR (TI "midwestern us" OR AB "midwestern us") OR (TI "midwestern state*" OR AB "midwestern state*") OR (TI "midwest state*" OR AB "midwest state*") OR (TI "midwest us" OR AB "midwest us") OR (TI "great plains" OR AB "great plains") OR (TI heartland OR AB heartland) OR (TI "new england" OR AB "new england") OR (TI "northeastern us" OR AB "northeastern us") OR (TI "northeastern state*" OR AB "northeastern state*") OR (TI "northeast state*" OR AB "northeast state*") OR (TI "northeast us" OR AB "northeast us") OR (TI "pacific northwest" OR AB "pacific northwest") OR (TI "northwestern us" OR AB "northwestern us") OR (TI northwest* AND TI "us") OR (AB northwest* AND AB "us") OR (TI "northwestern state*" OR AB "northwestern state*") OR (TI "northwest state*" OR AB "northwest state*") OR (TI "pacific state*" OR AB "pacific state*") OR (TI "southeast state*" OR AB "southeast state*") OR (TI "southeastern state*" OR AB "southeastern state*") OR (TI "southeast region" OR AB "southeast region") OR (TI "southeastern region" OR AB "southeastern region") OR (TI "southeast us" OR AB "southeast us") OR (TI "southeastern us" OR AB "southeastern us") OR (TI "southern state*" OR AB "southern state*") OR (TI "southern us" OR AB "southern us") OR (TI "southwest state*" OR AB "southwest state*") OR (TI "southwestern state*" OR AB "southwestern state*") OR (TI "southwest us" OR AB "southwest us") OR (TI "southwestern us" OR AB "southwestern us") OR (TI "deep south" OR AB "deep south") OR (TI "black belt" OR AB "black belt") OR (TI "rust belt" OR AB "rust belt") OR (TI "district of Columbia" OR AB "district of Columbia") OR (TI "Washington dc" OR AB "Washington dc") OR (TI Alabama OR AB Alabama) OR (TI Alaska OR AB Alaska) OR (TI Arizona OR AB Arizona) OR (TI Arkansas OR AB Arkansas) OR (TI California OR AB California) OR (TI Colorado OR AB Colorado) OR (TI Connecticut OR AB Connecticut) OR (TI Delaware OR AB Delaware) OR (TI Florida OR AB Florida) OR (TI Georgia OR AB Georgia) OR (TI Hawaii OR AB Hawaii) OR (TI "Hawai i" OR AB "Hawai i") OR (TI Idaho OR AB Idaho) OR (TI Illinois OR AB Illinois) OR (TI Indiana OR AB Indiana) OR (TI Iowa OR AB Iowa) OR (TI Kansas OR AB Kansas) OR (TI Kentucky OR AB Kentucky) OR (TI Louisiana OR AB Louisiana) OR (TI Maine OR AB Maine) OR (TI Maryland OR AB Maryland) OR (TI Massachusetts OR AB Massachusetts) OR (TI Michigan OR AB Michigan) OR (TI Minnesota OR AB Minnesota) OR (TI Mississippi OR AB Mississippi) OR (TI Missouri OR AB Missouri) OR (TI Montana OR AB Montana) OR (TI Nebraska OR AB Nebraska) OR (TI Nevada OR AB Nevada) OR (TI "New Hampshire" OR AB "New Hampshire") OR (TI "New Jersey" OR AB "New Jersey") OR (TI "New Mexico" OR AB "New Mexico") OR (TI "New York" OR AB "New York") OR (TI "North Carolina" OR AB "North Carolina") OR (TI "North Dakota" OR AB "North Dakota") OR (TI Ohio OR AB Ohio) OR (TI Oklahoma OR AB Oklahoma) OR (TI Oregon OR AB Oregon) OR (TI Pennsylvania OR AB Pennsylvania) OR (TI "Rhode Island" OR AB "Rhode Island") OR (TI "South Carolina" OR AB "South Carolina") OR (TI "South Dakota" OR AB "South Dakota") OR (TI Tennessee OR AB Tennessee) OR (TI Texas OR AB Texas) OR (TI Utah OR AB Utah) OR (TI Vermont OR AB Vermont) OR (TI Virginia OR AB Virginia) OR (TI Washington OR AB Washington) OR (TI "West Virginia" OR AB "West Virginia") OR (TI Wisconsin OR AB Wisconsin) OR (TI Wyoming OR AB Wyoming) OR (TI America* OR AB America*) OR (TI "Western Hemisphere" OR AB "Western Hemisphere"))))

2	2cv	CDoH: controlled vocabulary	DE(Taxes OR Marketing OR "Social Media" OR "Food Industry" OR Taxation OR "Marketing" OR "Advertising" OR "Digital Marketing" OR "Retailing" OR "Social Marketing" OR "Television Advertising" OR "Electronic Retailing" OR "Social Media" OR "Online Social Networks" OR "Social Media Influencers")
3	2ft	CDoH: free text	XB("marketing" OR advertis* OR "price promoti*" OR "food environment*" OR "food desert*" OR corporation* OR "corporate social responsibility" OR (corporat* and CSR) OR lobbying OR "social media" OR (facebook or twitter or tweet* or instagram or snapchat or youtube or tiktok or "viral content" or meme*) OR (online and influencer*) OR "working condition" OR ((labor or work* or industr*) and union*) OR monopol* OR (oligopoly or oligopolies) OR philanthrop* OR capitalis* OR neoliberal* OR "political economy" OR "market concentration" OR "civil society capture" OR "think tank*" OR "grocery industry")
4	2px	CDoH: proximity operator terms	XB((commercial N2 determinant*) OR (industr* N5 influenc*) OR (digital* N2 engage*) OR (suppress* N1 wage*) OR (price* N2 manipulat*) OR (tax* N5 (avoid* or credit* or dodg* or strateg*)) OR (brand* N2 (environment* or social or green*)) OR (food N3 retail*))
5	2combined	CDoH: combined	S2 OR S3 OR S4
6	3cv	Sugar-sweetened beverages (USDA NESR): controlled vocabulary	DE(Beverages OR "Carbonated Beverages" OR "Fruit and Vegetable Juices" OR Coffee OR Tea OR "Beverages (Nonalcoholic)" OR "Energy Drink" OR "Energy Drinks")
7	3ft	Sugar-sweetened beverages (USDA NESR): free text	XB(beverage* OR "caloric drink*" OR "sport* drink*" OR "protein drink*" OR "fortified drink*" OR "sweetened drink*" OR "sweet drink*" OR "sugary drink*" OR "dairy drink*" OR "chocolate drink*" OR "nutritional drink*" OR smoothie* OR "protein shake*" OR "meal replacement*" OR "carbonated drink*" OR "soft drink*" OR soda* OR "caffeinated drink*" OR "energy drink*" OR "sugar sweetened drink*" OR juice* OR "fruit drink*" OR "fizzy drink*" OR coffee* OR tea OR ((drink* or beverage* or diet*) and SSB*))
8	3combined	Sugar-sweetened beverages (USDA NESR): combined	S6 OR S7
9		Complete search strategy	S1 AND S5 AND S8
10		Filter: document type	Include: Academic Journals, Dissertations

Table S3. Search strategy & translations

Database: Food Science Technology Abstracts

Platform: EBSCOhost

Temporal Coverage: Database inception to December 12, 2025

#	ID	Explanatory Note	Search Terms
1	1	MLA search hedge: Latinx/Hispanic US Population	((TI Hispanic* OR AB Hispanic*) OR (TI hispano* OR AB hispano*) OR (TI hispana* OR AB hispana) OR (TI Hispaniola OR AB Hispaniola) OR (TI Latino* OR AB Latino*) OR (TI Latina* OR AB Latina*) OR (TI Latine* OR AB Latine*) OR (TI Latinu* OR AB Latinu*) OR (TI Latinx OR AB Latinx) OR (TI "Spanish American*" OR AB "Spanish American*") OR (TI "Mexican American*" OR AB "Mexican American*") OR (TI Chicana* OR AB Chicana*) OR (TI Chicano* OR AB Chicano*) OR (TI Chicanx OR AB Chicanx) OR (TI "Puerto Rico" OR AB "Puerto Rico") OR (TI "Puerto Rican*" OR AB "Puerto Rican*") OR (TI Boricua* OR AB Boricua*) OR (TI "Cuban American*" OR AB "Cuban American*") OR (((TI "Spanish speak*" OR AB "Spanish speak*") OR (TI Argentina OR AB Argentina) OR (TI Argentine* OR AB Argentine*) OR (TI Argentinian* OR AB Argentinian*) OR (TI Bolivia OR AB Bolivia) OR (TI Bolivian* OR AB Bolivian*) OR (TI Brazil OR AB Brazil) OR (TI Brazilian* OR AB Brazilian*) OR (TI Chile OR AB Chile) OR (TI Chilean* OR AB Chilean*) OR (TI Colombia OR AB Colombia) OR (TI Colombian* OR AB Colombian*) OR (TI "Costa Rica" OR AB "Costa Rica") OR (TI "Costa Rican*" OR AB "Costa Rican*") OR (TI Cub OR AB Cuba) OR (TI Cuban* OR AB Cuban*) OR (TI Dominican* OR AB Dominican*) OR (TI Ecuador OR AB Ecuador) OR (TI Ecuadorian* OR AB Ecuadorian*) OR (TI Ecuadorean* OR AB Ecuadorean*) OR (TI "El Salvador" OR AB "El Salvador") OR (TI Salvadorean* OR AB Salvadorean*) OR (TI Salvadoran* OR AB Salvadoran*) OR (TI Salvadorian* OR AB Salvadorian*) OR (TI Guatemala OR AB Guatemala) OR (TI Guatemalan* OR AB Guatemalan*) OR (TI Honduras OR AB Honduras) OR (TI Honduran* OR AB Honduran*) OR (TI Mexico OR AB Mexico) OR (TI Mexican* OR AB Mexican*) OR (TI Nicaragua OR AB Nicaragua) OR (TI Nicaraguan* OR AB Nicaraguan*) OR (TI Panama OR AB Panama) OR (TI Panamanian* OR AB Panamanian*) OR (TI Paraguay OR AB Paraguay) OR (TI Paraguayan* OR AB Paraguayan*) OR (TI Peru OR AB Peru) OR (TI Peruvian* OR AB Peruvian*) OR (TI Uruguay OR AB Uruguay) OR (TI Uruguayan* OR AB Uruguayan*) OR (TI Venezuela OR AB Venezuela) OR (TI Venezuelan* OR AB Venezuelan*) OR (TI "Central America*" OR AB "Central America*") OR (TI "Spanish Caribbean*" OR AB "Spanish Caribbean*") OR (TI "South America*" OR AB "South America*") OR (TI "Latin America*" OR AB "Latin America*")) AND ((TI "united state*" OR AB "united state*") OR (TI USA OR AB USA) OR (TI Appalachia* OR AB Appalachia*) OR (TI "great lakes" OR AB "great lakes") OR (TI "mid atlantic state*" OR AB "mid atlantic state*") OR (TI "mid atlantic region*" OR AB "mid atlantic region*") OR (TI "middle atlantic state*" OR AB "middle atlantic state*") OR (TI "middle atlantic region*" OR AB "middle atlantic region*") OR (TI "midwestern us" OR AB "midwestern us") OR (TI "midwestern state*" OR AB "midwestern state*") OR (TI "midwest state*" OR AB "midwest state*") OR (TI "midwest us" OR AB "midwest us") OR (TI "great plains" OR AB "great plains") OR (TI heartland OR AB heartland) OR (TI "new england" OR AB "new england") OR (TI "northeastern us" OR AB "northeastern us") OR (TI "northeastern state*" OR AB "northeastern state*") OR (TI "northeast state*" OR AB "northeast state*") OR (TI "northeast us" OR AB "northeast us") OR (TI "pacific northwest" OR AB "pacific northwest") OR (TI "northwestern us" OR AB "northwestern us") OR (TI northwest* AND TI "us") OR (AB northwest* AND AB "us") OR (TI "northwestern state*" OR AB "northwestern state*") OR (TI "northwest state*" OR AB "northwest state*") OR (TI "pacific state*" OR AB "pacific state*") OR (TI "southeast state*" OR AB "southeast state*") OR (TI "southeastern state*" OR AB "southeastern state*") OR (TI "southeast region" OR AB "southeast region") OR (TI "southeastern region" OR AB "southeastern region") OR (TI "southeast us" OR AB "southeast us") OR (TI "southeastern us" OR AB "southeastern us") OR (TI "southern state*" OR AB "southern state*") OR (TI "southern us" OR AB "southern us") OR (TI "southwest state*" OR AB "southwest state*") OR (TI "southwestern state*" OR AB "southwestern state*") OR (TI "southwest us" OR AB "southwest us") OR (TI "southwestern us" OR AB "southwestern us") OR (TI "deep south" OR AB "deep south") OR (TI "black belt" OR AB "black belt") OR (TI "rust belt" OR AB "rust belt") OR (TI "district of Columbia" OR AB "district of Columbia") OR (TI "Washington dc" OR AB "Washington dc") OR (TI Alabama OR AB Alabama) OR (TI Alaska OR AB Alaska) OR (TI Arizona OR AB Arizona) OR (TI Arkansas OR AB Arkansas) OR (TI California OR AB California) OR (TI Colorado OR AB Colorado) OR (TI Connecticut OR AB Connecticut) OR (TI Delaware OR AB Delaware) OR (TI Florida OR AB Florida) OR (TI Georgia OR AB Georgia) OR (TI Hawaii OR AB Hawaii) OR (TI "Hawai i" OR AB "Hawai i") OR (TI Idaho OR AB Idaho) OR (TI Illinois OR AB Illinois) OR (TI Indiana OR AB Indiana) OR (TI Iowa OR AB Iowa) OR (TI Kansas OR AB Kansas) OR (TI Kentucky OR AB Kentucky) OR (TI Louisiana OR AB Louisiana) OR (TI Maine OR AB Maine) OR (TI Maryland OR AB Maryland) OR (TI Massachusetts OR AB Massachusetts) OR (TI Michigan OR AB Michigan) OR (TI Minnesota OR AB Minnesota) OR (TI Mississippi OR AB Mississippi) OR (TI Missouri OR AB Missouri) OR (TI Montana OR AB Montana) OR (TI Nebraska OR AB Nebraska) OR (TI Nevada OR AB Nevada) OR (TI "New Hampshire" OR AB "New Hampshire") OR (TI "New Jersey" OR AB "New Jersey") OR (TI "New Mexico" OR AB "New Mexico") OR (TI "New York" OR AB "New York") OR (TI "North Carolina" OR AB "North Carolina") OR (TI "North Dakota" OR AB "North Dakota") OR (TI Ohio OR AB Ohio) OR (TI Oklahoma OR AB Oklahoma) OR (TI Oregon OR AB Oregon) OR (TI Pennsylvania OR AB Pennsylvania) OR (TI "Rhode Island" OR AB "Rhode Island") OR (TI "South Carolina" OR AB "South Carolina") OR (TI "South Dakota" OR AB "South Dakota") OR (TI Tennessee OR AB Tennessee) OR (TI Texas OR AB Texas) OR (TI Utah OR AB Utah) OR (TI Vermont OR AB Vermont) OR (TI Virginia OR AB Virginia) OR (TI Washington OR AB Washington) OR (TI "West Virginia" OR AB "West Virginia") OR (TI Wisconsin OR AB Wisconsin) OR (TI Wyoming OR AB Wyoming) OR (TI America* OR AB America*) OR (TI "Western Hemisphere" OR AB "Western Hemisphere"))))

2	2cv	CDoH: controlled vocabulary	DE(Taxation OR "MARKETING" OR "ADVERTISING" OR "Social Media")
3	2ft	CDoH: free text	TI("marketing" OR advertis* OR "price promoti*" OR "food environment*" OR "food desert*" OR corporation* OR "corporate social responsibility" OR (corporat* and CSR) OR lobbying OR "social media" OR (facebook or twitter or tweet* or instagram or snapchat or youtube or tiktok or "viral content" or meme*) OR (online and influencer*) OR "working condition" OR ((labor or work* or industr*) and union*) OR monopol* OR (oligopoly or oligopolies) OR philanthrop* OR capitalis* OR neoliberal* OR "political economy" OR "market concentration" OR "civil society capture" OR "think tank*" OR "grocery industry") OR AB("marketing" OR advertis* OR "price promoti*" OR "food environment*" OR "food desert*" OR corporation* OR "corporate social responsibility" OR (corporat* and CSR) OR lobbying OR "social media" OR (facebook or twitter or tweet* or instagram or snapchat or youtube or tiktok or "viral content" or meme*) OR (online and influencer*) OR "working condition" OR ((labor or work* or industr*) and union*) OR monopol* OR (oligopoly or oligopolies) OR philanthrop* OR capitalis* OR neoliberal* OR "political economy" OR "market concentration" OR "civil society capture" OR "think tank*" OR "grocery industry")
4	2px	CDoH: proximity operator terms	TI((commercial N2 determinant*) OR (industr* N5 influenc*) OR (digital* N2 engage*) OR (suppress* N1 wage*) OR (price* N2 manipulat*) OR (tax* N5 (avoid* or credit* or dodg* or strateg*)) OR (brand* N2 (environment* or social or green*)) OR (food N3 retail*)) OR AB((commercial N2 determinant*) OR (industr* N5 influenc*) OR (digital* N2 engage*) OR (suppress* N1 wage*) OR (price* N2 manipulat*) OR (tax* N5 (avoid* or credit* or dodg* or strateg*)) OR (brand* N2 (environment* or social or green*)) OR (food N3 retail*))
5	2combined	CDoH: combined	S2 OR S3 OR S4
6	3cv	Sugar-sweetened beverages (USDA NESR): controlled vocabulary	DE("BEVERAGE CONCENTRATES" OR "BEVERAGE MIXES" OR "BEVERAGE POWDERS" OR "BEVERAGES INDUSTRY" OR "CALORIES LOW BEVERAGES" OR "CANNED BEVERAGES" OR "CHILLED BEVERAGES" OR "CHOCOLATE BEVERAGES" OR "COCOA BEVERAGES" OR "COFFEE" OR "DAIRY BEVERAGES" OR "FLAVOURED BEVERAGES" OR "FORTIFIED BEVERAGES" OR "FROZEN BEVERAGES" OR "FUNCTIONAL BEVERAGES" OR "HEALTH BEVERAGES" OR "HONEY BEVERAGES" OR "HORCHATA" OR "INSTANT BEVERAGES" OR "LITE BEVERAGES" OR "READY TO DRINK BEVERAGES" OR "SOFT DRINKS" OR "SUGAR LOW BEVERAGES" OR "SWEETENED BEVERAGES" OR "TEAS" OR "SOFT DRINKS" OR "CARBONATED BEVERAGES" OR "COCA-COLA" OR "COLA BEVERAGES" OR "CHINOTTO" OR "ENERGY DRINKS" OR "FRUIT CORDIALS" OR "FRUIT JUICE BEVERAGES" OR "GINGER ALE" OR "GINGER BEER" OR "GUARANA" OR "LEMONADE" OR "ORANGE BEVERAGES" OR "PERFORMANCE DRINKS" OR "ROOT BEER" OR "SMOOTHIES" OR "SPORTS DRINKS" OR "SQUASH" OR "SARSAPARILLA (BEVERAGE)" OR "TONIC WATERS" OR "VEGETABLE JUICE BEVERAGES" OR "Ginseng Beverages" OR "Isotonic Drinks" OR "Sports Drinks" OR Juices OR "Fruit Juices" OR "Vegetable Juices")
7	3ft	Sugar-sweetened beverages (USDA NESR): free text	TI(beverage* OR "caloric drink*" OR "sport* drink*" OR "protein drink*" OR "fortified drink*" OR "sweetened drink*" OR "sweet drink*" OR "sugary drink*" OR "dairy drink*" OR "chocolate drink*" OR "nutritional drink*" OR smoothie* OR "protein shake*" OR "meal replacement*" OR "carbonated drink*" OR "soft drink*" OR soda* OR "caffeinated drink*" OR "energy drink*" OR "sugar sweetened drink*" OR juice* OR "fruit drink*" OR "fizzy drink*" OR coffee* OR tea OR ((drink* or beverage* or diet*) and SSB*)) OR AB(beverage* OR "caloric drink*" OR "sport* drink*" OR "protein drink*" OR "fortified drink*" OR "sweetened drink*" OR "sweet drink*" OR "sugary drink*" OR "dairy drink*" OR "chocolate drink*" OR "nutritional drink*" OR smoothie* OR "protein shake*" OR "meal replacement*" OR "carbonated drink*" OR "soft drink*" OR soda* OR "caffeinated drink*" OR "energy drink*" OR "sugar sweetened drink*" OR juice* OR "fruit drink*" OR "fizzy drink*" OR coffee* OR tea OR ((drink* or beverage* or diet*) and SSB*))
8	3combined	Sugar-sweetened beverages (USDA NESR): combined	S6 OR S7
9		Complete search strategy	S1 AND S5 AND S8
10		Filter: document type	Include: Academic Journals, Theses

Table S4. Search strategy & translations

Database: AGRICOLA

Platform: EBSCOhost

Temporal Coverage: Database inception to December 12, 2025

#	ID	Explanatory Note	Search Terms
1	1	MLA search hedge: Latinx/Hispanic US Population	(DE Latinos OR DE "Hispanic Americans" OR DE "Mexican Americans" OR DE "Puerto Ricans" OR DE "Cuban Americans" OR (TI Hispanic* OR AB Hispanic*) OR (TI hispano* OR AB hispano*) OR (TI hispana* OR AB hispana) OR (TI Hispaniola OR AB Hispaniola) OR (TI Latino* OR AB Latino*) OR (TI Latina* OR AB Latina*) OR (TI Latine* OR AB Latine*) OR (TI Latinu* OR AB Latinu*) OR (TI Latinx OR AB Latinx) OR (TI "Spanish American*" OR AB "Spanish American*") OR (TI "Mexican American*" OR AB "Mexican American*") OR (TI Chicana* OR AB Chicana*) OR (TI Chicano* OR AB Chicano*) OR (TI Chicanx OR AB Chicanx) OR (TI "Puerto Rico" OR AB "Puerto Rico") OR (TI "Puerto Rican*" OR AB "Puerto Rican*") OR (TI Boricua* OR AB Boricua*) OR (TI "Cuban American*" OR AB "Cuban American*") OR (((TI "Spanish speak*" OR AB "Spanish speak*") OR (TI Argentina OR AB Argentina) OR (TI Argentine* OR AB Argentine*) OR (TI Argentinian* OR AB Argentinian*) OR (TI Bolivia OR AB Bolivia) OR (TI Bolivian* OR AB Bolivian*) OR (TI Brazil OR AB Brazil) OR (TI Brazilian* OR AB Brazilian*) OR (TI Chile OR AB Chile) OR (TI Chilean* OR AB Chilean*) OR (TI Colombia OR AB Colombia) OR (TI Colombian* OR AB Colombian*) OR (TI "Costa Rica" OR AB "Costa Rica") OR (TI "Costa Rican*" OR AB "Costa Rican*") OR (TI Cub OR AB Cuba) OR (TI Cuban* OR AB Cuban*) OR (TI Dominican* OR AB Dominican*) OR (TI Ecuador OR AB Ecuador) OR (TI Ecuadorian* OR AB Ecuadorian*) OR (TI Ecuadorean* OR AB Ecuadorean*) OR (TI "El Salvador" OR AB "El Salvador") OR (TI Salvadorean* OR AB Salvadorean*) OR (TI Salvadoran* OR AB Salvadoran*) OR (TI Salvadorian* OR AB Salvadorian*) OR (TI Guatemala OR AB Guatemala) OR (TI Guatemalan* OR AB Guatemalan*) OR (TI Honduras OR AB Honduras) OR (TI Honduran* OR AB Honduran*) OR (TI Mexico OR AB Mexico) OR (TI Mexican* OR AB Mexican*) OR (TI Nicaragua OR AB Nicaragua) OR (TI Nicaraguan* OR AB Nicaraguan*) OR (TI Panama OR AB Panama) OR (TI Panamanian* OR AB Panamanian*) OR (TI Paraguay OR AB Paraguay) OR (TI Paraguayan* OR AB Paraguayan*) OR (TI Peru OR AB Peru) OR (TI Peruvian* OR AB Peruvian*) OR (TI Uruguay OR AB Uruguay) OR (TI Uruguayan* OR AB Uruguayan*) OR (TI Venezuela OR AB Venezuela) OR (TI Venezuelan* OR AB Venezuelan*) OR (TI "Central America*" OR AB "Central America*") OR (TI "Spanish Caribbean*" OR AB "Spanish Caribbean*") OR (TI "South America*" OR AB "South America*") OR (TI "Latin America*" OR AB "Latin America*")) AND ((TI "united state*" OR AB "united state*") OR (TI USA OR AB USA) OR (TI Appalachia* OR AB Appalachia*) OR (TI "great lakes" OR AB "great lakes") OR (TI "mid atlantic state*" OR AB "mid atlantic state*") OR (TI "mid atlantic region*" OR AB "mid atlantic region*") OR (TI "middle atlantic state*" OR AB "middle atlantic state*") OR (TI "middle atlantic region*" OR AB "middle atlantic region*") OR (TI "midwestern us" OR AB "midwestern us") OR (TI "midwestern state*" OR AB "midwestern state*") OR (TI "midwest state*" OR AB "midwest state*") OR (TI "midwest us" OR AB "midwest us") OR (TI "great plains" OR AB "great plains") OR (TI heartland OR AB heartland) OR (TI "new england" OR AB "new england") OR (TI "northeastern us" OR AB "northeastern us") OR (TI "northeastern state*" OR AB "northeastern state*") OR (TI "northeast state*" OR AB "northeast state*") OR (TI "northeast us" OR AB "northeast us") OR (TI "pacific northwest" OR AB "pacific northwest") OR (TI "northwestern us" OR AB "northwestern us") OR (TI northwest* AND TI "us") OR (AB northwest* AND AB "us") OR (TI "northwestern state*" OR AB "northwestern state*") OR (TI "northwest state*" OR AB "northwest state*") OR (TI "pacific state*" OR AB "pacific state*") OR (TI "southeast state*" OR AB "southeast state*") OR (TI "southeastern state*" OR AB "southeastern state*") OR (TI "southeast region" OR AB "southeast region") OR (TI "southeastern region" OR AB "southeastern region") OR (TI "southeast us" OR AB "southeast us") OR (TI "southeastern us" OR AB "southeastern us") OR (TI "southern state*" OR AB "southern state*") OR (TI "southern us" OR AB "southern us") OR (TI "southwest state*" OR AB "southwest state*") OR (TI "southwestern state*" OR AB "southwestern state*") OR (TI "southwest us" OR AB "southwest us") OR (TI "southwestern us" OR AB "southwestern us") OR (TI "deep south" OR AB "deep south") OR (TI "black belt" OR AB "black belt") OR (TI "rust belt" OR AB "rust belt") OR (TI "district of Columbia" OR AB "district of Columbia") OR (TI "Washington dc" OR AB "Washington dc") OR (TI Alabama OR AB Alabama) OR (TI Alaska OR AB Alaska) OR (TI Arizona OR AB Arizona) OR (TI Arkansas OR AB Arkansas) OR (TI California OR AB California) OR (TI Colorado OR AB Colorado) OR (TI Connecticut OR AB Connecticut) OR (TI Delaware OR AB Delaware) OR (TI Florida OR AB Florida) OR (TI Georgia OR AB Georgia) OR (TI Hawaii OR AB Hawaii) OR (TI "Hawai i" OR AB "Hawai i") OR (TI Idaho OR AB Idaho) OR (TI Illinois OR AB Illinois) OR (TI Indiana OR AB Indiana) OR (TI Iowa OR AB Iowa) OR (TI Kansas OR AB Kansas) OR (TI Kentucky OR AB Kentucky) OR (TI Louisiana OR AB Louisiana) OR (TI Maine OR AB Maine) OR (TI Maryland OR AB Maryland) OR (TI Massachusetts OR AB Massachusetts) OR (TI Michigan OR AB Michigan) OR (TI Minnesota OR AB Minnesota) OR (TI Mississippi OR AB Mississippi) OR (TI Missouri OR AB Missouri) OR (TI Montana OR AB Montana) OR (TI Nebraska OR AB Nebraska) OR (TI Nevada OR AB Nevada) OR (TI "New Hampshire" OR AB "New Hampshire") OR (TI "New Jersey" OR AB "New Jersey") OR (TI "New Mexico" OR AB "New Mexico") OR (TI "New York" OR AB "New York") OR (TI "North Carolina" OR AB "North Carolina") OR (TI "North Dakota" OR AB "North Dakota") OR (TI Ohio OR AB Ohio) OR (TI Oklahoma OR AB Oklahoma) OR (TI Oregon OR AB Oregon) OR (TI Pennsylvania OR AB Pennsylvania) OR (TI "Rhode Island" OR AB "Rhode Island") OR (TI "South Carolina" OR AB "South Carolina") OR (TI "South Dakota" OR AB "South Dakota") OR (TI Tennessee OR AB Tennessee) OR (TI Texas OR AB Texas) OR (TI Utah OR AB Utah) OR (TI Vermont OR AB Vermont) OR (TI Virginia OR AB Virginia) OR (TI Washington OR AB Washington) OR (TI "West Virginia" OR AB "West Virginia") OR (TI Wisconsin OR AB Wisconsin) OR (TI Wyoming OR AB Wyoming) OR (TI America* OR AB America*) OR (TI "Western Hemisphere" OR AB "Western Hemisphere"))))

2	2cv	CDoH: controlled vocabulary	(DE Taxes OR Marketing OR Advertising OR "Social Networks")
3	2ft	CDoH: free text	TI("marketing" OR advertis* OR "price promoti*" OR "food environment*" OR "food desert*" OR corporation* OR "corporate social responsibility" OR (corporat* and CSR) OR lobbying OR "social media" OR (facebook or twitter or tweet* or instagram or snapchat or youtube or tiktok or "viral content" or meme*) OR (online and influencer*) OR "working condition" OR ((labor or work* or industr*) and union*) OR monopol* OR (oligopoly or oligopolies) OR philanthrop* OR capitalis* OR neoliberal* OR "political economy" OR "market concentration" OR "civil society capture" OR "think tank*" OR "grocery industry") OR AB("marketing" OR advertis* OR "price promoti*" OR "food environment*" OR "food desert*" OR corporation* OR "corporate social responsibility" OR (corporat* and CSR) OR lobbying OR "social media" OR (facebook or twitter or tweet* or instagram or snapchat or youtube or tiktok or "viral content" or meme*) OR (online and influencer*) OR "working condition" OR ((labor or work* or industr*) and union*) OR monopol* OR (oligopoly or oligopolies) OR philanthrop* OR capitalis* OR neoliberal* OR "political economy" OR "market concentration" OR "civil society capture" OR "think tank*" OR "grocery industry")
4	2px	CDoH: proximity operator terms	TI((commercial N2 determinant*) OR (industr* N5 influenc*) OR (digital* N2 engage*) OR (suppress* N1 wage*) OR (price* N2 manipulat*) OR (tax* N5 (avoid* or credit* or dodg* or strateg*)) OR (brand* N2 (environment* or social or green*)) OR (food N3 retail*)) OR AB((commercial N2 determinant*) OR (industr* N5 influenc*) OR (digital* N2 engage*) OR (suppress* N1 wage*) OR (price* N2 manipulat*) OR (tax* N5 (avoid* or credit* or dodg* or strateg*)) OR (brand* N2 (environment* or social or green*)) OR (food N3 retail*))
5	2combined	CDoH: combined	S2 OR S3 OR S4
6	3cv	Sugar-sweetened beverages (USDA NESR): controlled vocabulary	DE("Sugar Sweetened Beverages" OR "Energy Drinks" OR "Fruit Drinks" OR "Soft Drinks" OR "Sports Drinks" OR "Carbonated Beverages" OR "Instant Coffee" OR Tea OR "Iced Tea")
7	3ft	Sugar-sweetened beverages (USDA NESR): free text	TI(beverage* OR "caloric drink*" OR "sport* drink*" OR "protein drink*" OR "fortified drink*" OR "sweetened drink*" OR "sweet drink*" OR "sugary drink*" OR "dairy drink*" OR "chocolate drink*" OR "nutritional drink*" OR smoothie* OR "protein shake*" OR "meal replacement*" OR "carbonated drink*" OR "soft drink*" OR soda* OR "caffeinated drink*" OR "energy drink*" OR "sugar sweetened drink*" OR juice* OR "fruit drink*" OR "fizzy drink*" OR coffee* OR tea OR ((drink* or beverage* or diet*) and SSB*)) OR AB(beverage* OR "caloric drink*" OR "sport* drink*" OR "protein drink*" OR "fortified drink*" OR "sweetened drink*" OR "sweet drink*" OR "sugary drink*" OR "dairy drink*" OR "chocolate drink*" OR "nutritional drink*" OR smoothie* OR "protein shake*" OR "meal replacement*" OR "carbonated drink*" OR "soft drink*" OR soda* OR "caffeinated drink*" OR "energy drink*" OR "sugar sweetened drink*" OR juice* OR "fruit drink*" OR "fizzy drink*" OR coffee* OR tea OR ((drink* or beverage* or diet*) and SSB*))
8	3combined	Sugar-sweetened beverages (USDA NESR): combined	S6 OR S7
9		Complete search strategy	S1 AND S5 AND S8
10		Filter: document type	Include: Academic Journals

Table S5. Search strategy & translations

Database: AGRICOLA

Platform: EBSCOhost

Temporal Coverage: Database inception to December 12, 2025

#	ID	Explanatory Note	Search Terms
1	1	MLA search hedge: Latinx/Hispanic US Population	(TS=(Hispanic* OR hispano* OR hispana* OR Latino* OR Latina* OR Latine* OR Latinu* OR Latinx OR "Spanish American*" OR "Mexican American*" OR Chicana* OR Chicano* OR Chicanx OR "Puerto Rico" OR "Puerto Rican*" OR Boricua* OR "Cuban American*") OR (TS=("Spanish speak*" OR Argentina OR Argentine* OR Argentinian* OR Bolivia OR Bolivian* OR Brazil OR Brazilian* OR Chile OR Chilean* OR Colombia OR Colombian* OR "Costa Rica" OR "Costa Rican*" OR Cuban* OR "Hispaniola" OR "Dominican Republic" OR Dominican* OR Ecuador OR Ecuadorian* OR Ecuadorean* OR "El Salvador" OR Salvadorean* OR Salvadoran* OR Salvadorian* OR Guatemala OR Guatemalan* OR Honduras OR Honduran* OR Mexico OR Mexican* OR Nicaraguan* OR Panama OR Panamanian* OR Paraguay OR Paraguayan* OR Peru OR Peruvian* OR Uruguay OR Uruguayan* OR Venezuela OR Venezuelan* OR "Central America*" OR "Caribbean Region" OR "Caribbean*" OR "South America" OR "South America*" OR "Latin America*") AND TS=("United State*" OR "USA" OR "North America" OR Appalachia* OR "great lakes" OR "mid atlantic state*" OR "mid atlantic region*" OR "middle atlantic state*" OR "middle atlantic region*" OR "midwestern us" OR "midwestern state*" OR "midwest state*" OR "midwest us" OR "great plains" OR "heartland" OR "new england" OR "northeastern us" OR "northeastern state*" OR "northeast state*" OR "northeast us" OR "pacific northwest" OR (northwest* AND “us") OR "northwestern state*" OR "northwest state*" OR "pacific state*" OR "southeast state*" OR "southeastern state*" OR "southeast us" OR "southeastern us" OR "southern state*" OR "southern us" OR "southwest state*" OR "southwestern state*" OR "southwest us" OR "southwestern us" OR "deep south" OR "black belt" OR "rust belt" OR "District of Columbia" OR "Washington DC" OR Alabama OR Alaska OR Arizona OR Arkansas OR California OR Colorado OR Connecticut OR Delaware OR Florida OR Georgia OR Hawaii OR "Hawai'i" OR Idaho OR Illinois OR Indiana OR Iowa OR Kansas OR Kentucky OR Louisiana OR Maine OR Maryland OR Massachusetts OR Michigan OR Minnesota OR Mississippi OR Missouri OR Montana OR Nebraska OR Nevada OR "New Hampshire" OR "New Jersey" OR "New Mexico" OR "New York" OR "North Carolina" OR "North Dakota" OR Ohio OR Oklahoma OR Oregon OR Pennsylvania OR "Rhode Island" OR "South Carolina" OR "South Dakota" OR Tennessee OR Texas OR Utah OR Vermont OR Virginia OR Washington OR "West Virginia" OR Wisconsin OR Wyoming OR "Western Hemisphere" OR "Americas" OR America*)))
2	2ft	CDoH: free text	TS=("marketing" OR advertis* OR "price promoti*" OR "food environment*" OR "food desert*" OR corporation* OR "corporate social responsibility" OR (corporat* and CSR) OR lobbying OR "social media" OR (facebook or twitter or tweet* or instagram or snapchat or youtube or tiktok or "viral content" or meme*) OR (online and influencer*) OR "working condition" OR ((labor or work* or industr*) and union*) OR monopol* OR (oligopoly or oligopolies) OR philanthrop* OR capitalis* OR neoliberal* OR "political economy" OR "market concentration" OR "civil society capture" OR "think tank*" OR "grocery industry")
3	2px	CDoH: proximity operator terms	TS=((commercial NEAR/2 determinant*) OR (industr* NEAR/5 influenc*) OR (digital* NEAR/2 engage*) OR (suppress* NEAR/1 wage*) OR (price* NEAR/2 manipulat*) OR (tax* NEAR/5 (avoid* or credit* or dodg* or strateg*)) OR (brand* NEAR/2 (environment* or social or green*)) OR (food NEAR/3 retail*))
4	2combined	CDoH: combined	#2 OR #3
5	3	Sugar-sweetened beverages (USDA NESR): free text	TS=(beverage* OR "caloric drink*" OR "sport* drink*" OR "protein drink*" OR "fortified drink*" OR "sweetened drink*" OR "sweet drink*" OR "sugary drink*" OR "dairy drink*" OR "chocolate drink*" OR "nutritional drink*" OR smoothie* OR "protein shake*" OR "meal replacement*" OR "carbonated drink*" OR "soft drink*" OR soda* OR "caffeinated drink*" OR "energy drink*" OR "sugar sweetened drink*" OR juice* OR "fruit drink*" OR "fizzy drink*" OR coffee* OR tea OR ((drink* or beverage* or diet*) and SSB*))
6		Complete search strategy	#1 AND #4 AND #5
7		Filter: document type	Include: Article, Proceeding Paper, Review Article, Early Access

Table S6. Preferred Reporting Items for Systematic reviews and Meta-Analyses extension for Scoping Reviews (PRISMA-ScR) Checklist

SECTION	ITEM	PRISMA-ScR CHECKLIST ITEM	REPORTED ON PAGE #
**TITLE**			
Title	1	Identify the report as a scoping review.	1
**ABSTRACT**			
Structured summary	2	Provide a structured summary that includes (as applicable): background, objectives, eligibility criteria, sources of evidence, charting methods, results, and conclusions that relate to the review questions and objectives.	1-2
**INTRODUCTION**			
Rationale	3	Describe the rationale for the review in the context of what is already known. Explain why the review questions/objectives lend themselves to a scoping review approach.	2-3
Objectives	4	Provide an explicit statement of the questions and objectives being addressed with reference to their key elements (e.g., population or participants, concepts, and context) or other relevant key elements used to conceptualize the review questions and/or objectives.	3
**METHODS**			
Protocol and registration	5	Indicate whether a review protocol exists; state if and where it can be accessed (e.g., a Web address); and if available, provide registration information, including the registration number.	3
Eligibility criteria	6	Specify characteristics of the sources of evidence used as eligibility criteria (e.g., years considered, language, and publication status), and provide a rationale.	3-4
Information sources*	7	Describe all information sources in the search (e.g., databases with dates of coverage and contact with authors to identify additional sources), as well as the date the most recent search was executed.	4
Search	8	Present the full electronic search strategy for at least 1 database, including any limits used, such that it could be repeated.	4
Selection of sources of evidence†	9	State the process for selecting sources of evidence (i.e., screening and eligibility) included in the scoping review.	4
Data charting process‡	10	Describe the methods of charting data from the included sources of evidence (e.g., calibrated forms or forms that have been tested by the team before their use, and whether data charting was done independently or in duplicate) and any processes for obtaining and confirming data from investigators.	4-5
Data items	11	List and define all variables for which data were sought and any assumptions and simplifications made.	3-4
Critical appraisal of individual sources of evidence§	12	If done, provide a rationale for conducting a critical appraisal of included sources of evidence; describe the methods used and how this information was used in any data synthesis (if appropriate).	4
Synthesis of results	13	Describe the methods of handling and summarizing the data that were charted.	4-5
**RESULTS**			
Selection of sources of evidence	14	Give numbers of sources of evidence screened, assessed for eligibility, and included in the review, with reasons for exclusions at each stage, ideally using a flow diagram.	5
Characteristics of sources of evidence	15	For each source of evidence, present characteristics for which data were charted and provide the citations.	6-7
Critical appraisal within sources of evidence	16	If done, present data on critical appraisal of included sources of evidence (see item 12).	7
Results of individual sources of evidence	17	For each included source of evidence, present the relevant data that were charted that relate to the review questions and objectives.	6-9

SECTION	ITEM	PRISMA-ScR CHECKLIST ITEM	REPORTED ON PAGE #
Synthesis of results	18	Summarize and/or present the charting results as they relate to the review questions and objectives.	9-10
**DISCUSSION**			
Summary of evidence	19	Summarize the main results (including an overview of concepts, themes, and types of evidence available), link to the review questions and objectives, and consider the relevance to key groups.	10-11
Limitations	20	Discuss the limitations of the scoping review process.	11-12
Conclusions	21	Provide a general interpretation of the results with respect to the review questions and objectives, as well as potential implications and/or next steps.	12
**FUNDING**			
Funding	22	Describe sources of funding for the included sources of evidence, as well as sources of funding for the scoping review. Describe the role of the funders of the scoping review.	12

JBI = Joanna Briggs Institute; PRISMA-ScR = Preferred Reporting Items for Systematic reviews and Meta-Analyses extension for Scoping Reviews.
* Where *sources of evidence* (see second footnote) are compiled from, such as bibliographic databases, social media platforms, and Web sites.
† A more inclusive/heterogeneous term used to account for the different types of evidence or data sources (e.g., quantitative and/or qualitative research, expert opinion, and policy documents) that may be eligible in a scoping review as opposed to only studies. This is not to be confused with *information sources* (see first footnote).
‡ The frameworks by Arksey and O'Malley (6) and Levac and colleagues (7) and the JBI guidance (4, 5) refer to the process of data extraction in a scoping review as data charting.
§ The process of systematically examining research evidence to assess its validity, results, and relevance before using it to inform a decision. This term is used for items 12 and 19 instead of "risk of bias" (which is more applicable to systematic reviews of interventions) to include and acknowledge the various sources of evidence that may be used in a scoping review (e.g., quantitative and/or qualitative research, expert opinion, and policy document).

From: Tricco AC, Lillie E, Zarin W, O'Brien KK, Colquhoun H, Levac D, et al. PRISMA Extension for Scoping Reviews (PRISMAScR): Checklist and Explanation. Ann Intern Med. 2018;169:467–473. doi: 10.7326/M18-0850.
